# Increased production of soluble CTLA-4 in patients with spondylarthropathies correlates with disease activity

**DOI:** 10.1186/ar2747

**Published:** 2009-07-01

**Authors:** Éric Toussirot, Philippe Saas, Marina Deschamps, Fabienne Pouthier, Lucille Perrot, Sylvain Perruche, Jacqueline Chabod, Pierre Tiberghien, Daniel Wendling

**Affiliations:** 1 Department of Rheumatology, University Hospital Jean Minjoz, Bd Fleming 25030 Besançon cedex, France; 2 EA 3186 «Agents Pathogènes et Inflammation» University of Franche-Comté, IFR133, Place St Jacques, Besançon, France; 3 CIC-Biotherapy 506, University Hospital St-Jacques, Place St Jacques, 25030 Besançon cedex, France; 4 INSERM UMR645, University of Franche-Comté, EFS Bourgogne Franche-Comté, Plateforme de Biomonitoring, CIC-Biotherapy 506, IFR133, Bd Fleming, 25020 Besançon cedex, France; 5 EFS Bourgogne Franche-Comté, Bd Fleming, 25020 Besançon cedex, France

## Abstract

**Introduction:**

Spondylarthropathies (SpA) are characterized by abnormal immune responses including T cell activation. Cytotoxic T lymphocyte associated molecule-4 (CTLA-4) is involved in down-regulating immune responses. A soluble form of CTLA-4 (sCTLA-4), resulting from an alternative splicing, has been identified and was found increased in several autoimmune diseases. Here, we evaluated circulating levels of sCTLA-4 as a marker of immune dysregulation in SpA. Intracellular CTLA-4 and levels of *CTLA-4 *transcript expression in peripheral blood lymphocytes (PBL) were also studied.

**Methods:**

Sera from 165 patients with SpA were evaluated for sCTLA-4 measurements. Results were compared with those from 71 patients with rheumatoid arthritis (RA) and 88 healthy subjects. In 32 patients with SpA, 22 patients with RA and 15 healthy controls, we analyzed the intracellular CTLA-4 expression in CD4+ T cells, CD8+ T cells, activated (HLA-DR+Foxp3-) CD4+ T cells, CD4+ regulatory (CD25+Foxp3+) T cells and in CD3 negative cells by flow cytometry. Expression of the full length (coding for membrane CTLA-4) and spliced form (coding for sCTLA-4) of *CTLA-4 *transcripts in PBL were analyzed by quantitative real-time polymerase chain reaction (QRT-PCR).

**Results:**

High levels of sCTLA-4 were found in the SpA group compared to the RA group and healthy controls (*P *< 0.0001). Soluble CTLA-4 serum levels strongly correlated with clinical index of disease activity BASDAI (r = 0.42, *P *< 0.0001) and C-reactive protein (CRP) levels (r = 0.17, *P *= 0.037). In contrast to RA patients, SpA patients did not exhibit changes in intracellular CTLA-4 expression in the different PBL subsets tested. Finally, the SpA group showed a preferential expression of the spliced *CTLA-4 *mRNA (*P *= 0.0014) in PBL.

**Conclusions:**

SpA patients exhibit high levels of circulating sCTLA-4 that may result from an alternative splicing of *CTLA-4 *transcripts. This may influence immune activation and regulation in SpA.

## Introduction

Seronegative spondylarthropathies (SpA) are chronic inflammatory diseases mainly affecting the axial skeleton and leading to progressive spinal ankylosis. Peripheral arthritis and enthesopathic lesions are also observed. The prototype of the disease is ankylosing spondylitis (AS), and other clinical features include reactive arthritis, psoriatic arthritis, enteropathic arthritis and undifferentiated SpA [1]. All these diseases have a common genetic background, namely the Histocompatibility Leukocyte Antigen (HLA) Class I B27, which is strongly associated to SpA and particularly to AS.

The pathophysiology of these diseases is not completely understood, but it is believed that the genetic component is directly involved as demonstrated by transgenic rats expressing human HLA-B27 [2]. Environmental factors also play a role in SpA, particularly bacterial antigens from the gut or the genitourinary tract [3]. Different cellular subsets are observed in the joints or entheseal structures of SpA including macrophages and neutrophils, and also CD8+ and CD4+ T lymphocytes [4,5]. It has been suggested that aberrant immune response occurs in SpA or that SpA results from molecular mimicry. It is thought that unregulated CD8+ and/or CD4+ T cell responses to bacterial or self antigens participate in SpA pathophysiology and that both responses involve the HLA-B27 molecule [6]. Indeed, CD4+T cells are required for the development of the disease in the animal model of transgenic rat for human HLA-B27.

CD28 expressed on T cells and its ligands, CD80 and CD86, present on the antigen presenting cells (APC) are the prototypical costimulatory molecules [7]. Cytotoxic T lymphocyte associated molecule-4 (CTLA-4 or CD152) is expressed on T cells after activation, but it is constitutively expressed by CD4+CD25+ regulatory cells (Treg) [8]. This molecule is homologous to CD28 and binds to CD80 and CD86 with a higher affinity. Membrane bound CTLA-4 acts by interfering with CD28/CD80-CD86 interactions by competitive binding and also by mediating a negative signal interfering with T cell receptor stimulation [9]. Thus, CTLA-4 triggering leads to a down regulation of T cell response, induction of apoptosis and/or immunological anergy [7]. On the other hand, CTLA-4 is absolutely required for the suppressive function of naturally occurring FoxP3+ Treg, at least in mice [10]. In addition to CD4+ and CD8+ T cells, B cells also express CTLA-4 [8].

A soluble form of CTLA-4 (sCTLA-4) has also been described [11,12]. This soluble molecule results from an alternative transcript of the *CTLA-4 *gene, which lacks the third exon encoding the transmembrane domain of the CTLA-4 molecule [11,12]. This soluble form, detected in CD4+ and CD8+ T cells, B cells and lymphoid organs [8], is able to bind CD80 and CD86. Recent data showed the increase of sCTLA-4 in the serum of patients with various autoimmune diseases, such as thyroiditis [13], myasthenia gravis [14], systemic lupus erythematosus (SLE) [15,16], and systemic sclerosis [17]. It has been suggested that sCTLA-4 may interfere with normal immune response, favoring autoreactivity in these conditions [11]. In addition, genetic studies revealed that there is an association between *CTLA-4 *gene polymorphism and susceptibility to autoimmune diseases [18].

Abnormal T cell activation is observed in SpA, suggesting a possible defect of lymphocyte inhibitory molecules. Thus, here, we investigated the serum levels of sCTLA-4 in a large cohort of patients with SpA. Then, we analyzed the cellular expression of CTLA-4 on different peripheral blood lymphocyte (PBL) subsets, as well as *CTLA-4 *transcripts in PBL. The results obtained in SpA patients were compared with those obtained in patients with rheumatoid arthritis (RA).

## Materials and methods

### Patients with spondylarthropathy

We enrolled 165 consecutive patients who met the European Spondylarthropathy Study group criteria for SpA [19] and were receiving follow-up at our department (Rheumatology Department, Besançon, France). Clinical assessment included the following demographic data: age, sex, disease duration, and extra-articular manifestations (uveitis). The clinical activity was evaluated using the Bath Ankylosing Spondylitis Disease Activity Index (BASDAI) [20] and the functional score Bath Ankylosing Spondylitis Functional Index (BASFI) [21]. Erythrocyte sedimentation rate (ESR), and C reactive protein (CRP) levels were used as laboratory parameters to assess inflammation. Biologic assessment also included HLA-B27 determination. All the patients received non-steroidal anti-inflammatory drugs (NSAIDs) and some of them had a second-line treatment such as sulfasalazine (n = 23) or methotrexate (n = 10). A limited number of patients had corticosteroids at a low dosage (daily prednisolone ≤ 10 mg; n = 8). In order to avoid anti-TNF-α agent as a confounding factor, patients under or who had previously received anti-TNF-α agent were excluded from this study.

### Patients with rheumatoid arthritis

Seventy-one patients with RA meeting 1987 American College of Rheumatology criteria [22] were included for purposes of comparison. For each of these patients, the following data were recorded: age; sex; disease duration; extra-articular manifestations (subcutaneous nodules, vasculitis, sicca syndrome, pulmonary, or cardiac involvement); tender and swollen joint counts; the Health Assessment Questionnaire (HAQ) score; the ESR and CRP levels; and whether tests were positive for rheumatoid factors.

### Patients with systemic lupus erythematosus

In order to have another comparative group (with previously determined elevated serum sCTLA-4 levels) [15,16], 14 patients with SLE (13 females, 1 male, mean age 44.1 ± 5.5 years) responding to the 1982 revised American Rheumatism Association criteria [23] were also recruited.

### Control subjects

The control group consisted of 88 healthy subjects without inflammatory conditions (platelets donors). HLA class I antigen determination was available in this group. As most of the patients with SpA were HLA-B27 positive and the frequency of this antigen in the normal population is around 8%, we selected 24 HLA-B27-positive subjects from the platelet donors for this control group.

All the patients and control subjects gave their informed consent to participate in the study according to the Helsinki declaration and our study protocol was approved by our local ethics committee (*comité d'éthique clinique du CHU de Besançon*).

### Determination of circulating soluble CTLA-4

Serum concentrations of sCTLA-4 in patients with SpA, RA, or SLE and control subjects were determined by ELISA using reagent kits for human sCTLA-4 (AbCys, Paris, France) according to the manufacturer's instructions.

### Flow cytometric analysis of intracellular expression of CTLA-4 on lymphocyte subsets

Absolute numbers of blood T cells, CD4+ and CD8+ T cells were determined by single platform flow cytometry using the TetraCXP^® ^method, Flow-Count fluorospheres, and FC500^® ^cytometer (Beckman Coulter, Villepinte, France) according to the manufacturer's recommendations [24]. CTLA-4 is not expressed on human naive resting T cells and is only expressed after activation on the surface at low levels (less than 10% of total CTLA-4) [8], so we decided to evaluate intracellular CTLA-4 expression in fixed and permeabilized lymphocytes to appreciate CTLA-4 expression in different lymphocyte subsets. Staining was performed on ethylenediaminetetraacetic acid (EDTA) venous blood samples after red blood cell lysis. Cells were stained with allophycocyanin-conjugated CD3, phycoerythrin-cyanin 7 (PE-Cy7)-conjugated CD4, fluorescein isothiocyanate (FITC)-conjugated CD8 antibodies (Beckman Coulter, Villepinte, France), then fixed, permeabilized and stained again using PE-conjugated anti-CTLA-4 (anti-CD152) antibody (Beckman Coulter, Villepinte, France) before being analyzed using FACS Canto II^® ^cytometer (BD Biosciences, Le Pont de Claix, France). The same experiments were also performed using conjugated anti-HLA-DR, FITC-conjugated anti-CD25, PE Texas Red (ECD) conjugated anti-CD69 (Beckman Coulter, Villepinte, France) or conjugated anti-FoxP3 (clone PCH101, eBioscience, San Diego, CA, USA) [25] antibodies. Intracellular expression of CTLA-4 was examined in CD3+ T cells, in activated (HLA-DR+) CD4+ T cells, in early activated (CD69+) CD4+ T cells, and in (CD25+ FoxP3+) CD4 + Treg. Non-specific staining was determined using labeled irrelevant control antibodies from the same antibody providers. Intracellular CTLA-4 analysis was performed in 32 SpA patients, 22 RA patients, and 15 healthy controls. These patients and healthy donors were randomly selected and corresponded to consecutive patients (samples were collected between August 2006 and April 2007).

### Quantification of full length and spliced CTLA-4 transcripts in peripheral blood mononuclear cells

The human CTLA-4 gene contains four exons. The full length *CTLA-4 *mRNA contains these four exons, encoding the CTLA-4 molecule expressed at the cell membrane [9]. sCTLA-4 is generated by alternatively spliced mRNA. This splicing event deletes the entire transmembrane region of the CTLA-4 molecule (corresponding to exon 3). Thus, the spliced *CTLA-4 *mRNA contains only three exons, encoding for the soluble form of CTLA-4 [11]. Total RNA was extracted from 3 × 10^6 ^of cells using the RNeasy^® ^Blood mini kit (QIAGEN, Courtaboeuf, France) according to the manufacturer's instructions, and reverse transcribed using random hexamers and M-MLV reverse transcriptase (Life Technologies, Rockville, MD, USA) to use as a template for quantitative real-time polymerase chain reaction (QRT-PCR). QRT-PCR reactions were performed in duplicate using gene-specific probes and a universal master mix (Applied Biosystems, Courtaboeuf, France) on an iCycler iQ thermocycler (Bio-Rad Laboratories, Marnes-la-Coquette, France). For full length *CTLA-4 *transcript analysis, gene-specific primers and bi-fluorescent hydrolysis probes were obtained from Assays-on-DemandTM gene expression products (#Hs99999101_m1; Applied Biosystems, Courtaboeuf, France). Expression of *spliced CTLA-4 *transcripts was analyzed using the following primer pair designed by ourselves (sense, anti-sense and probe respectively): 5'-CATCTGCAAGGTGGAGCTCAT-3', 5'-GGCTTCTTTTCTTTAGCAATTACATAAATC-3', and 5'-FAM-ACCGCCATACTACCTGGGCATAGGCA-TAMRA-3'. Analysis was carried out using the ΔΔCt method [26] with the pool of 15 healthy donor PBL samples as a calibrator and *18S *as a housekeeping reference gene. Target gene expression was expressed as a fold change compared with healthy donor PBL.

### Statistical analysis

Results were expressed as mean ± standard error of the mean (SEM; range). Only non-parametric tests were used. Statistical analysis between the three groups (healthy controls, SpA, and RA) involved non-parametric analysis of variance (ANOVA) using the Kruskal-Wallis test. This test was used to compare age, circulating sCTLA-4 levels, T cell subsets, and intracellular CTLA-4 expression. When a significant difference was found between the three groups, a two-group analysis was performed a second time (SpA *vs *controls, RA *vs *controls and SpA *vs *RA) using the Mann-Whitney *U*-test. This test was also used to compare variables (disease duration, ESR, CRP) between SpA and RA patients and between SLE patients and healthy controls (sCTLA-4). Qualitative data (sex) were analyzed using the chi-squared test. Wilcoxon test was used to compare the relative expression of the full length and the spliced form of *CTLA-4 *transcripts in patients with SpA and in patients with RA. Spearman's *r*-test was used to calculate correlations between sCTLA-4 and indices of disease activity of SpA or RA, and between *CTLA-4 *transcripts and sCTLA-4 in patients with SpA. Values of *P *less than 0.05 were considered significant.

## Results

### Study population

The demographic, clinical characteristics, and laboratory features of the patients with SpA, patients with RA and healthy controls (platelet donors) are reported in Table 1. Of the 165 patients with SpA, 117 had AS, 7 had reactive arthritis, 5 had psoriatic arthritis, 5 had enteropathic arthritis, and 31 had undifferentiated SpA. At the time of evaluation, extra-articular manifestations were present in 21% and peripheral arthritis in 27.8% of patients with SpA. The HLA-B27 antigen was detected in 87.8% of patients. Among RA patients, 36.6% had extra-articular organ involvement.

**Table 1:** Clinical characteristics and laboratory features of patients with SpA or RA and healthy controls

	**HC** **(n = 88)**	**SpA** **(n = 165)**	**RA** **(n = 71)**	**P**
**Age (years)**	44.4 ± 0.8 (20 to 62)	42.9 ± 1.1 (18 to 75)	59.1 ± 1.4 (19 to 80)	< 0.0001 (KW) SpA vs HC (MW): *P *= 0.28 RA vs SpA and RA vs HC (MW): *P *< 0.0001
**Sex**	56 M/16 F	121 M/44 F	27 M/44 F	SpA vs HC vs RA: *P *< 0.0001 (χ^2^) SpA vs HC: *P *= 0.9 RA vs SpA and RA vs HC: *P *< 0.0001 (χ^2^)
**Disease duration (years)**		9.2 ± 0.6 (0.5 to 35)	10.2 ± 1.1 (0.5 to 40)	0.5 (MW)
**Extra-articular manifestations (%)**		35/165 (21)	26/71 (36.6)	
**Peripheral arthritis (%)**		46/165 (27.8)		
**BASDAI (0 to 100)**		35.9 ± 2.2 (0.4 to 94)		
**BASFI (0 to 100)**		41.2 ± 2.9 (0 to 92)		
**Swollen joint count (0 to 28)**			7.2 ± 0.8 (0 to 22)	
**Tender joint count (0 to 28)**			4.7 ± 0.8 (0 to 28)	
**HAQ (0 to 3)**			1.5 ± 0.1 (0 to 3)	
**HLA-B27 (%)**		145 (87.8)		
**Rheumatoid factors (%)**			64/71 (90)	
**ESR (mm/h)**		28.2 ± 2.1 (1 to 163)	30.3 ± 3.1 (1 to 125)	0.3 (MW)
**CRP (mg/l)**		25.1 ± 3.2 (0 to 350)	22.3 ± 3.5 (1 to 132)	0.8 (MW)

Results are expressed as mean ± standard error of the mean (range). The Kruskal-Wallis test was used to compare age, disease duration, ESR, and CRP in the three groups. Then, Mann-Whitney *U*-test was used to calculate the exact *P *values between two groups.

BASDAI = Bath Ankylosing Spondylitis Disease Activity Index; BASFI = Bath Ankylosing Spondylitis Functional Index; CRP = C-reactive protein; ESR = erythrocyte sedimentation rate; HAQ = Health Assessment Questionnaire; HC = healthy controls; HLA = Histocompatibility Leukocyte Antigen; KW = Kruskal-Wallis; MW = Mann-Whitney; NS = not significant; RA = rheumatoid arthritis; SpA = spondylarthropathie; χ^2 ^= chi-squared test.

The three groups (SpA, RA, healthy controls) differed regarding age (*P *< 0.0001) and pairwise tests showed that the patients with RA were older compared with the patients with SpA or healthy controls (*P *< 0.0001). However, age was similar in the SpA group and the control group (*P *= 0.28). The proportion of female patients was higher in the RA group than in the SpA and control groups (*P *< 0.0001), whereas the sex ratio was the same in the SpA and control subjects (*P *= 0.9). No differences were found between SpA and RA for disease duration, ESR, or CRP levels (all *P *> 0.05).

### Analysis of circulating sCTLA-4

**Figure 1:**
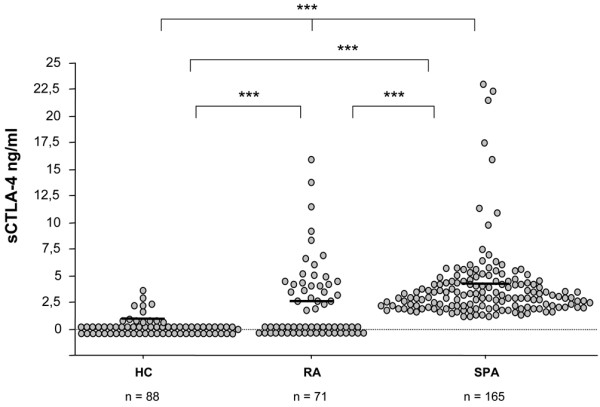
Serum concentrations of soluble Cytotoxic T Lymphocyte Antigen- 4 (sCTLA-4) in patients with spondylarthropathies (SpA, n = 165), with rheumatoid arthritis (RA, n = 71) and healthy controls (HC, n = 88). Circulating sCTLA-4 was measured by ELISA, as described in the Material & Methods section. Horizontal lines represent means (***: *P *< 0.005; statistical tests and exact p values are given in the Result section).

Serum sCTLA-4 levels were significantly higher in the SpA group (3.66 ± 0.3 ng/ml, 1 to 22.7) compared with the RA group (2.25 ± 0.4 ng/ml, 0 to 15.7) or the control group (0.25 ± 0.1 ng/ml, 0 to 3.4; Kruskal-Wallis: *P *< 0.0001). Serum sCTLA-4 levels were also found higher in patients with SLE (19.58 ± 2.7 ng/ml, 5.9 to 41) compared with healthy controls (Mann-Whitney *P *< 0.0001) or compared to patients with SpA or RA (Mann-Whitney *P *< 0.0001). Both SpA and RA patients had higher sCTLA-4 values compared with controls (Mann-Whitney *P *< 0.0001 and *P *= 0.001, respectively), whereas sCTLA-4 levels remained higher in the SpA group compared with RA (*P *< 0.0001; Figure 1).

**Figure 2:**
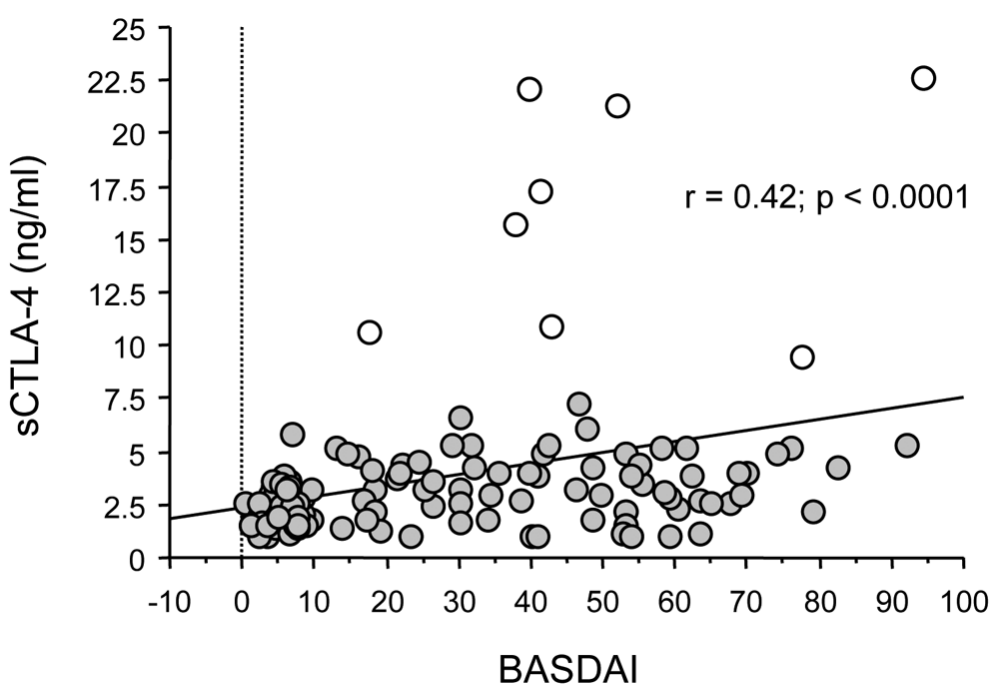
Correlation between soluble Cytotoxic T Lymphocyte Antigen- 4 (sCTLA-4) and clinical index of disease activity Bath Ankylosing Spondylitis Disease Activity Index in 165 patients with spondylarthropathies. The Bath Ankylosing Spondylitis Disease Activity Index (BASDAI) was evaluated as described in [19]. The statistical test used was Spearman's r test. When the eight extreme values (white circles) are not considered for correlation analysis, a significant correlation between sCTLA-4 and BASDAI score is still observed: (Spearman's test: r = 0.382, *P *< 0.0001).

In the SpA group, serum sCTLA-4 correlated with BASDAI (r = 0.42, *P *< 0.0001; Figure 2) and CRP (r = 0.17, *P *= 0.037) but not with ESR, BASFI, age, or disease duration (all *P *> 0.05). Serum sCTLA-4 levels were not influenced by the expression of the HLA-B27 antigen, the presence of extra-articular disease, or peripheral arthritis (all *P *> 0.05), whereas male patients had higher sCTLA-4 than female patients (Mann-Whitney: 3.97 ± 0.3, 1 to 22.7; *vs *2.84 ± 0.39, 1 to 17.3 ng/ml; *P *= 0.02). In addition, age was not correlated with serum sCTLA-4 levels in the different groups of subjects (all *P *> 0.05).

In the RA group, sCTLA-4 levels were correlated with swollen joint count (r = -0.25, *P *= 0.04) but not with laboratory parameters exploring inflammation (ESR and CRP), HAQ score, and tender joint score (all *P *> 0.05).

Finally, in normal subjects, we did not observe any significant differences in serum sCTLA-4 levels between male and female subjects or between HLA-B27-positive and HLA-B27-negative subjects (all *P *> 0.05).

To better understand this increase in circulating sCTLA-4 in patients with SpA, we then evaluated intracellular CTLA-4 expression. This analysis was performed in a representative number of patients and controls (i.e., 32 patients with SpA, 22 patients with RA, and 15 healthy controls).

### Analysis of blood lymphocyte subsets

Before analysis of intracellular CTLA-4 expression in lymphocyte subsets, we first compared absolute numbers of circulating CD3+, CD4+, and CD8+ T cells in the SpA group, in the RA group, and in healthy volunteers involved in this analysis. The three groups differed significantly regarding the number of CD3+ and CD8+ T cells (*P *≤ 0.005; Table 2). Indeed, RA patients had lower absolute numbers of CD3+ and CD8+ T cells compared with healthy controls or patients with SpA (*P *< 0.01 for each test). No difference in CD3+ T cells was observed between SpA patients and healthy controls, while CD8+ T cells were found to be lower in SpA, a result that was just near the significant level (*P *= 0.051).

**Table 2:** Absolute number of circulating CD3+ T cells, CD4+ T cells, CD8+ T cells, percentage ofactivated CD4+ T cells, regulatory CD4+ T cells (Treg) and percentage of cells expressing intracellular CTLA-4 in healthy controls, patients with Spondylarthropathies and rheumatoid arthritis

	**Healthy controls** **(n = 15)**	**Spondylarthropathies** **(n = 32)**	**Rheumatoid arthritis** **(n = 22)**	***P***
**CD3+ T cells (/mm^3^)**	1397 ± 79 (925 to 1806)	1360 ± 80.8 (622 to 2681)	1129 ± 140 (336 to 3024)	0.005 (KW) RA vs HC 0.004 (MW) SpA vs HC NS SpA vs RA 0.007 (MW)
**CD4+ T cells (/mm^3^)**	817 ± 45 (449 to 1024)	875 ± 49.5 (391 to 1699)	804 ± 102 (239 to 2306)	NS
**CD8+ T cells (/mm^3^)**	573 ± 47 (379 to 860)	457 ± 39.6 (130 to 979)	326 ± 52 (72 to 1260)	0.0006 (KW) RA vs HC 0.0007 (MW) SpA vs HC 0.051 RA vs SpA 0.006 (MW)
**HLA-DR+ Foxp3-** **CD4+ T cells** **(% of CD4+ T cells)**	16.6 ± 5.53 (5 to 70)	22.03 ± 4.56 (4 to 100)	12.14 ± 1.29 (5 to 26)	NS
**CD25+ Foxp3+** **CD4+ T cells** **(% of CD4+T cells)**	7.94 ± 1.04 (3.6 to 16)	8.2 ± 0.61 (4 to 14.4)	8.72 ± 0.7 (2.9 to 13.5)	NS
**Intracellular CTLA-4 in CD4+ T cells (%)**	4.35 ± 1.10 (1 to 13.3)	2.51 ± 0.40 (0 to 5.9)	1.35 ± 0.48^*,§^ (0 to 7.5)	0.005 (KW) RA vs HC 0.003 SpA vs HC NS RA vs SpA 0.02
**Intracellular CTLA-4 in CD4+ Treg (%)**	2.76 ± 0.44 (0.5 to 6.3)	1.88 ± 0.34 (0 to 5)	0.90 ± 0.32^*,§^ (0 to 3.9)	0.007 (KW) RA vs HC 0.0045 SpA vs HC NS SpA vs RA 0.027
**Intracellular CTLA-4 in Activated CD4+ T cells (%)**	1.59 ± 0.72 (0 to 7)	0.63 ± 0.26 (0 to 5)	0.5 ± 0.29 (0 to 5)	NS
**Intracellular CTLA-4 in CD8+ T cells (%)**	2.03 ± 0.57 (0.4 to 7)	2.64 ± 0.32 (0 to 6.3)	3.49 ± 0.88 (0.1 to 13.7)	NS
**Intracellular CTLA-4 in CD3- lymphocytes (%)**	0.89 ± 0.72 (0 to 8)	0.67 ± 0.21 (0 to 3.3)	3.50 ± 0.77^*,§^ (0 to 11)	0.0025 (KW) RA vs HC 0.0077 SpA vs HC NS SpA vs RA: 0.0017
**Intracellular CTLA-4 in the lymphocyte gate (total lymphocytes) (%)**	7.27 ± 1.27 (1.4 to 15)	3 ± 0.48 (0 to 7.6)	6.89 ± 1.3^§^ (0.1 to 19.3)	0.004 (KW) RA vs HC 0.0045 SpA vs HC NS SpA vs RA 0.007

Percentage of intracellular CTLA-4 was determined by flow cytometry. Results are expressed as mean ± standard error of the mean, (range). The Kruskal-Wallis (KW) test was used to compare T cell subsets and intracellular CTLA-4 expression in the three groups. Then, Mann-Whitney *U*-test was used to calculate the exact *P *values between two groups.

HC = healthy controls; HLA = Histocompatibility Leukocyte Antigen; KW = Kruskal-Wallis; MW = Mann-Whitney; NS = not significant; RA = rheumatoid arthritis; SpA = spondylarthropathie.

We then examined the percentage of circulating activated CD4+ T cells and Treg in the three studied groups. No difference was observed between these three groups for activated CD4+ T cells or for Treg (all *P *> 0.05; Table 2).

### Analysis of intracellular CTLA-4 expression in T cell subsets

**Figure 3:**
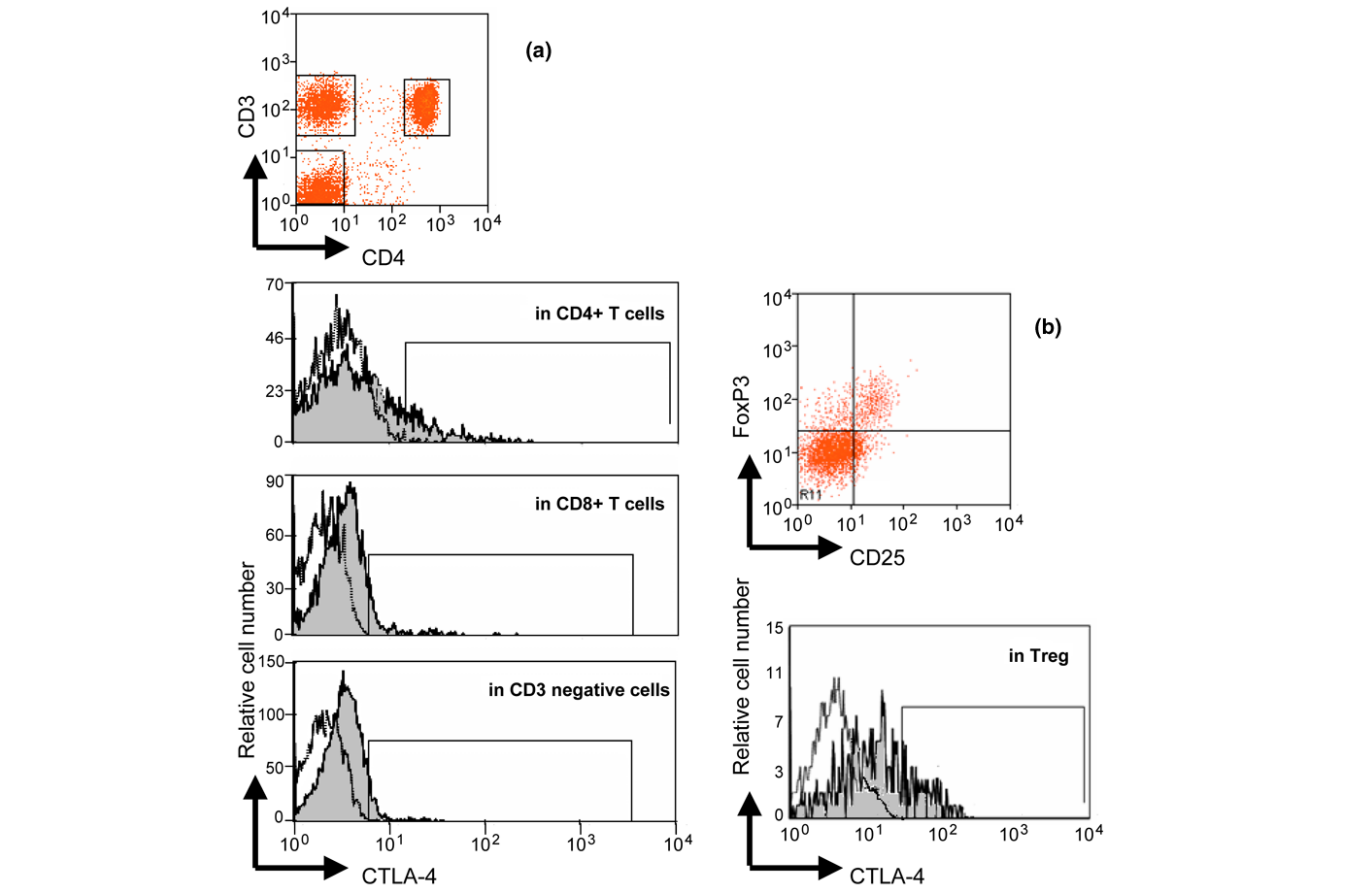
Representative analysis of intracellular Cytotoxic T Lymphocyte Antigen- 4 (CTLA-4) expression in peripheral blood lymphocyte subsets. This analysis was performed by flow cytometry as described in the Material & Methods section. **(a) **CD4+ T cells, CD8+ T cells and CD3- cells were identified in the PE-CY7 fluorescence vs allophycocyanin fluorescence gate based on the expression of CD3 and CD4 (CD4+ T cells, gate R6), of only CD3 (CD8+ T cells, gate R7), of neither CD3 nor CD4 (CD3- cells, gate R4). Then, intracellular expression of CTLA-4 was determined on these cell populations by comparing PE-conjugated isotype control antibody staining (open curves) with PE-conjugated anti-CD152 monoclonal antibody staining (gray filled curves). **(b) **Using the same gating strategy (CD3 vs CD4 dot plot), Treg were analyzed using FoxP3 *vs *CD25 dot plot. Intracellular CTLA-4 expression was also determined on CD3+ CD4+ CD25+ FoxP3+ cells by comparing PE-conjugated isotype control antibody staining (open curves) with PE-conjugated anti-CD152 (CTLA-4) monoclonal antibody staining (gray filled curves). The same analysis was performed for activated (CD3+ CD4+ HLA-DR+) T cells (data not shown). An example of intracellular CTLA-4 expression in CD4+ T cells, CD8+ T cells (defined as CD3+ CD4-), CD3- cells and Treg obtained with such gating strategy for a patient with spondylarthropathy is shown.

As shown in Table 2 and Figure 3, a significant difference was observed in the percentage of cells positive for intracellular CTLA-4 in CD4+ T cells, Treg, non T cells (CD3-negative cells in the lymphocyte gate, as assessed by flow cytometry; this presumably corresponds to B cells rather than natural killer cells [8]) and in the total lymphocyte gate between the three groups (*P *< 0.01 for each test), while no similar difference was found for activated CD4+ T cells or CD8+ T cells. However, SpA patients and healthy controls did not differ with regard to intracellular CTLA-4 in all these distinct lymphocyte subsets (all *P *> 0.05). Conversely, RA patients had lower intracellular CTLA-4 expression in CD4+ T cells and Treg compared with healthy controls or patients with SpA (all *P *< 0.05). The expression of intracellular CTLA-4 in CD3 negative cells (presumably B cells) was found to be higher in RA patients compared with healthy controls or SpA (*P *< 0.01). This increase of intracellular CTLA-4 in CD3-negative cells of RA patients leads to an increase of total intracellular CTLA-4 when considering all the lymphocytes (*P *= 0.004), while no difference was observed between SpA patients and healthy controls in these cells. Thus, increase of circulating sCTLA-4 in patients with SpA did not reflect an increase of intracellular CTLA-4 (independent on the examined T cell population). This prompted us to complete our analysis by evaluating the different *CTLA-4 *transcripts.

### Analysis of CTLA-4 full length and spliced mRNA transcripts

**Figure 4:**
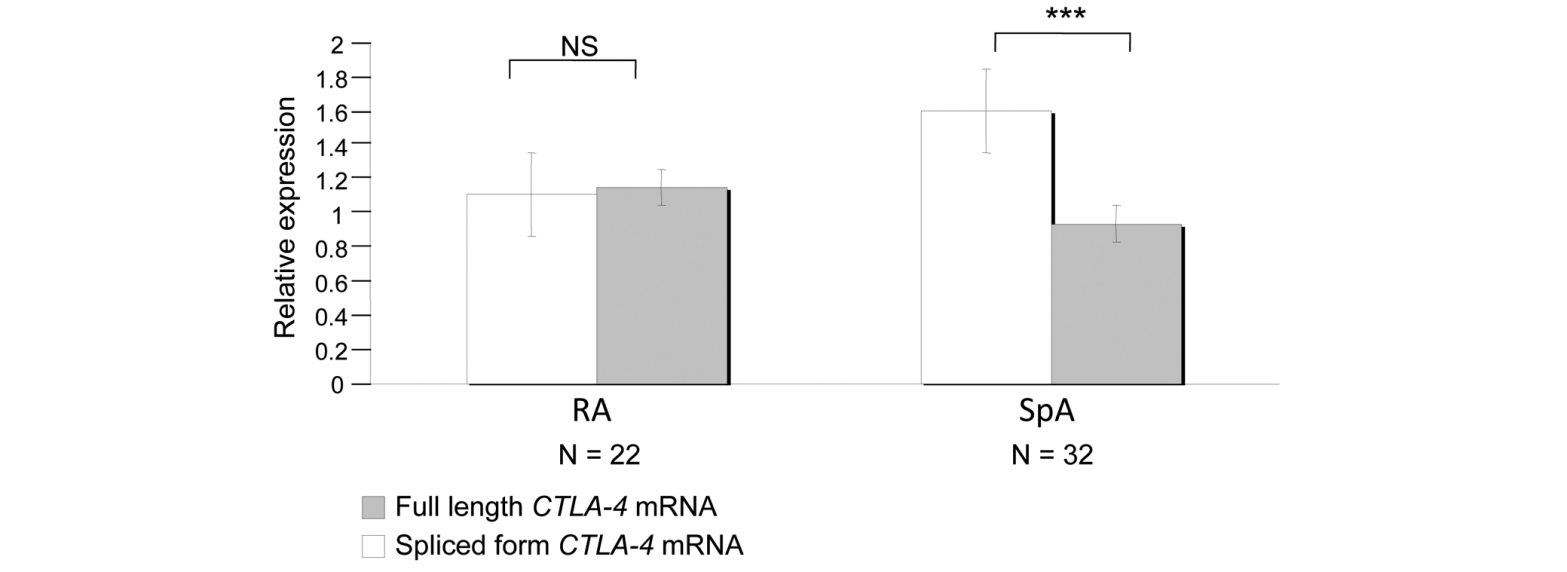
Relative expression of the 2 *Cytotoxic T lymphocyte Antigen-4 (CTLA-4) *transcripts, the full length form and the spliced form in peripheral blood lymphocytes of patients with spondylarthropathies (SpA) and patients with rheumatoid arthritis (RA). The full length form (gray bars) contains four exons and codes for the CTLA-4 molecule expressed at the cell membrane while the spliced form (open bars) contains three exons, lacks the transmembrane region and codes for the soluble CTLA-4 molecule [8]. Results (mean ± standard error of the mean) are obtained using the ΔΔCt method and expressed as a fold change of each *CTLA-4 *transcript comparative with the reference obtained with the pool of 15 healthy donor PBL. Wilcoxon test was used to compare the relative production of the full length and the spliced form in patients with SpA and in patients with RA (*** *P *< 0.005). NS = not significant.

**Figure 5:**
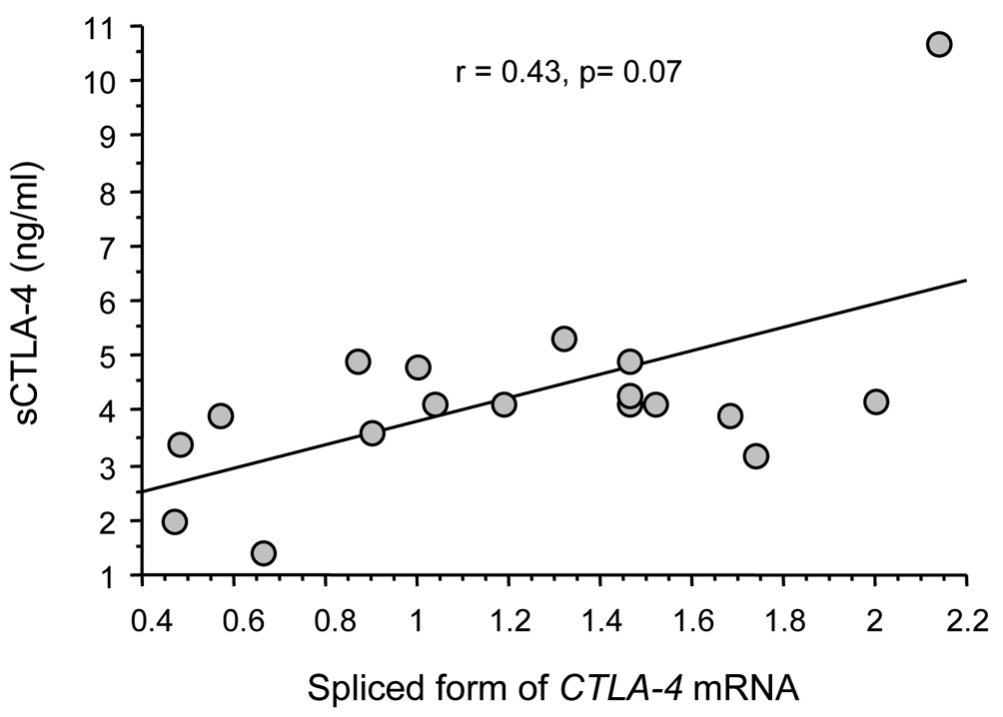
Correlation between serum concentrations of soluble Cytotoxic T Lymphocyte Antigen-4 (sCTLA-4, expressed in ng/ml) and the spliced form of *CTLA-4 *transcript expression (spliced form of the *CTLA-4 *mRNA, analyzed in PBL and expressed as a fold change comparative with the reference obtained with the pool of 15 healthy donor PBL). This spliced form of the *CTLA-4 *transcript encodes for the soluble CTLA-4 molecule. The statistical test used was Spearman's r test.

The pool of PBL from the 15 healthy donors was used as a reference. In patients with SpA, we observed an increase expression of the spliced *CTLA-4 *mRNA transcript (mean ± SEM, range, 1.608 ± 0.2, 0.09 to 4.14) relative to the full form (Figure 4; mean ± SEM, range, 0.942 ± 0.11, 0.03 to 2.3, *P *= 0.0014). Conversely, there was no difference in the expression of the full length form *versus *the spliced form in patients with RA (mean ± SEM, range, 1.117 ± 0.28, 0.1 to 2.7 for the spliced form transcript *vs*. 1.157 ± 0.4, 0.13 to 4.59 for the full length transcript expression, *P *= 0.7). We also found a tendency for a correlation between spliced *CTLA-4 *mRNA transcript levels and serum sCTLA-4 in patients with SpA (r = 0.43, *P *= 0.07; Figure 5). No similar correlation was observed in the RA group. Altogether, these data suggest that the increase of sCTLA-4 found in SpA patient serum could be related to an increase of spliced *CTLA-4 *mRNA transcripts.

## Discussion

Here, we evaluated circulating sCTLA-4 as a marker of disease activity for SpA. The role of sCTLA-4 is still not completely understood in contrast to membrane-bound CTLA-4, a negative regulator of T cell functions [8,9]. sCTLA-4 – resulting from an alternative transcript of the *CTLA-4 *gene [11,12] – is also able to bind CD80 and CD86 and may prevent membrane-bound CTLA-4 or CD28 interactions with their ligands [12]. Thus, sCTLA-4 may interfere with both (CD28-mediated) activating and (CTLA-4-mediated) inhibitory signaling pathways. The role of sCTLA-4 in T cell responses and inflammatory diseases have been recently highlighted by the description of the association of *CTLA-4 *gene polymorphisms (49 G/G and CT60 G/G) with the risk for common autoimmune diseases, such as Graves' disease, autoimmune hypothyroidism, and type I diabetes [18]. The *CTLA-4 *CT60G/G genotype was associated with lower *sCTLA-4 *transcript abundance in CD4+ T cells [17]. Despite the absence of sCTLA-4 protein analysis, these data by Ueda and colleagues [18] suggest that reduced sCTLA-4 (at least in CD4+ T cells) is associated with increased T cell activation and thereby autoreactivity.

On the contrary, further studies on circulating sCTLA-4-analyzed by ELISA- in several autoimmune diseases showed high levels of serum sCTLA-4 in patients with Graves' disease [13], Hashimoto thyroiditis [13], myasthenia gravis [14], SLE [15,16], and systemic sclerosis [17]. In SLE, elevated serum sCTLA-4 levels were correlated with the clinical index of disease activity SLEDAI in one study [16], but not in a second [15]. In systemic sclerosis, patients exhibited high levels of serum sCTLA-4 that correlated with disease activity [16]. In addition, sCTLA-4 was associated with cutaneous extension of fibrosis and tended to decrease in parallel to skin sclerosis improvement in a longitudinal analysis of some patients [17]. Taken together, these results suggest that high levels of circulating sCTLA-4 are observed in several autoimmune diseases and can be associated with disease severity and activity. This argues for a role of sCTLA-4 in enhancing T cell activation or preventing T cell regulation, leading finally to exacerbation of the disease [16,17].

The main finding of our study is that there are increased levels of serum sCTLA-4 in patients with SpA, compared with levels observed in patients with RA or in healthy volunteers. Circulating sCTLA-4 correlated with clinical index of disease activity BASDAI and CRP levels. As previously reported [12-15], serum sCTLA-4 was very low in our group of healthy controls, even in the 24 positive HLA-B27 subjects. We also confirm that patients with SLE had high circulating sCTLA-4 levels (around 70-fold more than control subjects). Our cohort of patients included mainly AS and undifferentiated SpA, but we did not observed significant differences between SpA subgroups (data not shown). As expected, the majority of patients with SpA expressed the HLA-B27 antigen, but again, this had no influence on the levels of sCTLA-4. In addition, no correlation was found between sCTLA-4, age, or the clinical characteristics of the patients. However, we found that male patients had higher sCTLA-4 levels than female patients but this may be explained by a predominance of male patients with SpA (73.3% in our series).

Our patients had conventional treatments for SpA (i.e. mainly NSAIDs) and a limited number of patients had a second-line treatment. However, it is considered that these treatments had no influence on T cell activation and costimulation pathways and therefore on the level of sCTLA-4. Eight SpA patients among the 165 analyzed here received oral prednisolone. Corticosteroids have been shown to decrease membrane CTLA-4 expression after human T cell activation [27]. Moreover, oral prednisolone in asthmatic patients has been shown to reduce serum sCTLA-4 concentrations after two weeks of treatment [28]. However, this can not be evaluated in our cohort because the number of patients on prednisolone is very low and concentrations are different (<10 mg/day in our study *vs *30 mg/day [28]). The impact of therapy on circulating sCTLA-4 is an interesting question to address.

The increase of sCTLA-4 in the serum of our patients with SpA contrasted with a normal percentage of cells positive for intracellular CTLA-4 (in comparison with the percentage found in healthy controls), suggesting a preferential expression of the spliced form of the *CTLA-4 *gene. Indeed, *CTLA-4 *mRNA analysis confirms this hypothesis because the spliced *CTLA-4 *RNA transcripts were found to be expressed at a higher level compared with the full length form. These results indicate that SpA patients had a high production of sCTLA-4 which was related to a preferential transcription of the *CTLA-4 *mRNA spliced form. The reason for the main expression of these *CTLA-4 *transcripts in SpA is not known but one may hypothesize that genetic background could play a role [18]. However, our cohort to date did not include a sufficient number of patients to appreciate the relation between circulating sCTLA-4, *CTLA-4 *mRNA spliced form, and *CTLA-4 *polymorphism.

SpAs are characterized by an infiltration of the sacroiliac joints, the synovium and entheseal structures by inflammatory cells. Different cells are found in these lesions [4,5]. Both CD4+ and CD8+ T lymphocytes are described at the pathological sites as well as B cells and macrophages [4,5]. These infiltrated T cells are activated and require the active participation of costimulation pathways. Thus, a high production of sCTLA-4 in SpA may potentially influence immune responses. As stated above, sCTLA-4 may block the down regulation of activated T cells mediated by CTLA-4-CD80/CD86 interactions, enhancing immune response *in situ*. Costimulation pathways have not been previously evaluated in SpA. However, in the HLA-B27 transgenic rat model of SpA, a decrease in formation of conjugates between dendritic cells expressing HLA-B27 and T cells was observed [29]. This decrease in dendritic cell-T cell conjugates was not related to a decrease of CD80 or CD86 expression, but resulted from a defective CD86 costimulatory pathway [29]. One may speculate that this defective APC function is due to the blockade of CD86 by sCTLA-4.

Treg are also implicated in the inflammatory response in SpA. Treg were found to be enriched in synovial tissues from patients with SpA, contrasting with a low percentage of Treg in the blood compartment [30]. In our study, we did not observe a significant reduction in the percentage of circulating Treg in our SpA patients as compared with healthy controls and RA patients. Treg constitutively expressed CTLA-4 [8] and suppressive functions of some Treg subsets are dependent on CTLA-4 [10,31]. Thus, one may speculate that a high production of sCTLA-4 in SpA may interfere with Treg functions, leading to a sustained and/or enhanced immune response. As stated above, the relation between sCTLA-4 and disease activity in different autoimmune diseases argues for a blockade of the inhibitory signal resulting from CTLA-4/CD80-CD86 binding.

In this study, serum sCTLA-4 was found mildly elevated in patients with RA compared with healthy controls. In addition, the percentage of cells positive for intracellular CTLA-4 was found decreased in CD4+ T cells and also in Treg. These results were not explained by the relative lymphopenia that characterized our RA patients, which involved CD3+ T cells and CD8+ T cells, while CD4+ T cells and Treg did not differ between patients and controls. On the other hand, intracellular CTLA-4 was found increased in the CD3-negative cell population, presumably B cells. Finally, there was no preferential expression of *CTLA-4 *transcripts in RA. Altogether, these results suggest that patients with RA had mild elevation of sCTLA-4 that may result from an increased expression of CTLA-4 in 'non T' cell subsets. RA is a chronic joint disease characterized by a major infiltration of the synovium by T cells. These cells are activated with the main implication of the CD28/CTLA-4 – CD80/CD86 ligand pairs. Altered cell surface expression of CD28, CTLA-4, CD80, and CD86 has been described in RA [32]. Thus, high levels of sCTLA-4 (whatever the producing cell was) may sustain T cell activation leading to inflammation and 'autoaggression', as observed in other autoimmune diseases. Reduced CTLA-4 expression in Treg can be related to defective function previously reported in RA [33,34]. A recent study reports that diminished CTLA-4 expression by Treg from RA patients was related to an increase rate of CTLA-4 internalization after activation [34].

## Conclusions

Similar to other autoimmune diseases [16,17], SpA are characterized by high serum sCTLA-4 levels with a significant and positive correlation with disease activity. These high levels of circulating sCTLA-4 in SpA were not explained by altered intracellular expression of CTLA-4, but by a preferential expression of the spliced form of *CTLA-4 *transcripts, a result that was not observed in RA. This suggests a potential immunologic role for sCTLA-4 in SpA. These results also indicate that sCTLA-4 could potentially be used as a surrogate marker for measuring disease activity in SpA.

Finally, the implication of sCTLA-4 in SpA also suggests the potential use of agents blocking costimulation pathways in this group of disorders. Indeed, a recent manuscript reports the use of abatacept – the fusion protein associating the CTLA-4 extracellular domain and the constant fragment region of human IgG1 and blocking CD28/CD80-CD86 costimulatory pathway- in a patient with undifferentiated SpA. After 12 months of treatment with abatacept, the patient was in complete remission [35].

## Abbreviations

ANOVA: analysis of variance; APC: antigen presenting cells; BASDAI: Bath Ankylosing Spondylitis Disease Activity Index; BASFI: Bath Ankylosing Spondylitis Functional Index; CRP: C-reactive protein; CTLA-4: cytotoxic T lymphocyte antigen-4; EDTA: ethylenediaminetetraacetic acid; ELISA: enzyme-linked immunosorbent assay; ESR: erythrocyte sedimentation rate; FITC: fluorescein isothiocyanate; HAQ: Health Assessment Questionnaire; HLA: Histocompatibility Leukocyte Antigen; NSAIDs: non-steroidal anti-inflammatory drugs; PBL: peripheral blood lymphocytes; Pe-cy 7: phycoerythrin-cyanin 7; RA: rheumatoid arthritis; sCTLA-4: soluble cytotoxic T lymphocyte antigen-4; SEM: standard error of the mean; SLE: systemic lupus erythematosus; SpA: spondylarthropathies; TNF: tumor necrosis factor; Treg: regulatory T cells.

## Competing interests

The authors declare that they have no competing interests.

## Authors' contributions

ET is the main investigator who conceived and designed the study and drafted the manuscript. He contributed to the recruitment of the patients involved in the study, analyzed the Results, and performed the statistical analysis. PS is responsible for laboratory parameter assessment. He contributed to the preparation of the manuscript by writing the Methods section. He analyzed and contributed to the discussion of the results. MD performed *CTLA-4 *transcript analysis, supervised sample collection, ELISA, and cytometry analysis. LP collected samples and performed flow cytometry experiments, ELISA. SP supervised cytometry and analyzed cytometry results. FP participated to the study by recruiting healthy controls (platelets donors) including HLA-B27 positive subjects. JC selected the HLA-B27 positive healthy donors among platelet donors. PT is the director of EFS Bourgogne Franche-Comté and of INSERM UMR645 University of Franche-Comté. He contributed to the discussion of the results. DW is the head of the Department of Rheumatology and contributed to the work as a clinical investigator. All authors read and approved the final manuscript.
